# Influence of the Co-Adsorbed Ions on the Surface-Enhanced Raman Scattering Spectra of Dopamine Adsorbed on Nanostructured Silver

**DOI:** 10.3390/ma15175972

**Published:** 2022-08-29

**Authors:** Aleksandra Michałowska, Kacper Jędrzejewski, Andrzej Kudelski

**Affiliations:** Faculty of Chemistry, University of Warsaw, Pasteura 1 Str., 02-093 Warsaw, Poland

**Keywords:** surface-enhanced Raman scattering, dopamine detection, nanostructured silver

## Abstract

The abnormal metabolism or imbalance of dopamine may lead to some neurological disorders. Therefore, the facile and fast detection of this neurotransmitter is essential in the early diagnosis of some diseases. One of the methods that can be used for the detection and determination of dopamine is the surface-enhanced Raman scattering (SERS). In this contribution, we report a very strong influence of some salts (we used salts containing Na^+^ cations and the following anions: SO_4_^2^^−^, F^−^, Cl^−^, Br^−^, and I^−^) on the spectral patterns and intensity of the SERS spectra of dopamine adsorbed on a nanostructured macroscopic silver substrate. The analysis of the recorded SERS spectra based on the assignments of Raman bands from the density-functional theory (DFT) calculations and based on the SERS surface selection rules reveals that when molecules of dopamine are adsorbed from an aqueous solution to which no electrolytes have been added, they adopt a flat orientation versus the silver surface; whereas, the molecules of dopamine co-adsorbed with various ions interact with the silver surface, mainly via phenolic groups, and they adopt a perpendicular orientation versus the metal surface. An addition of electrolytes also significantly influences the intensity of the recorded SERS spectrum; for example, an addition of Na_2_SO_4_ to a final concentration of 1 M induces an increase in the intensity of the measured SERS spectrum by a factor of ca. 40. This means that the addition of electrolytes to the analyzed solution can reduce the limit of detection of dopamine by SERS spectroscopy. The abovementioned findings may facilitate the construction of dopamine SERS sensors.

## 1. Introduction

**Figure 1:**
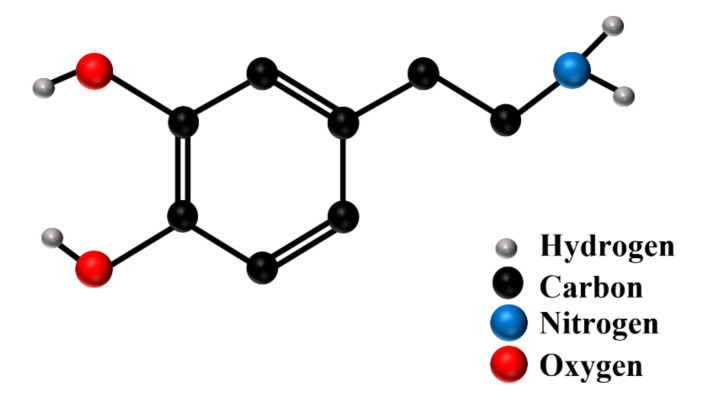
Structure of a dopamine molecule.

Dopamine (3,4-dihydroxyphenethylamine) is one of the most important neurotransmitters in the human body, and it belongs to the catecholamine family (the structure of the dopamine molecule is shown in Figure 1). Dopamine has a crucial role not only in the central nervous system but also in the hormonal, renal, and cardiovascular systems [1,2]. As a messenger substance, it is responsible for secretory, cognitive, and motor functions at the same time. Disturbed concentration levels of dopamine have been connected to various neurological disorders, such as Parkinson’s disease [3,4], Alzheimer’s disease [5], schizophrenia [6,7], or attention deficit hyperactivity disorder (ADHD) [8,9]. Dopamine is present in biological fluids in very low concentrations (0.01–1 μM) [10]; therefore, the sensitive detection of this neurotransmitter is necessary, for instance, in the diagnostics of diseases or in the control of medical treatments.

Many methods for detecting dopamine have been reported, including high-performance liquid chromatography (HPLC) [11,12], electrochemistry [13,14,15], fluorescence [16,17], and surface-enhanced Raman scattering (SERS) [18,19,20]. When comparing these techniques, very similar limits of detection (LOD) were obtained for electrochemical and fluorescence measurements as follows: 3.27 × 10^−7^ M [13] and 3 × 10^−7^ M [16], respectively. The LOD achieved in HPLC experiments is significantly lower and was estimated as being equal to 4.2 × 10^−9^ M [11]. However, the lowest LOD for dopamine identification (2.3 × 10^−10^ M) was achieved using SERS spectroscopy [18]. Moreover, chromatographic and fluorescence techniques require a long preparation time and they are usually quite complicated procedures [16,20]. In the case of an electrochemical detection of dopamine, there are also some limitations observed, mainly related to interference substances, such as uric acid or ascorbic acid [13].

In summary, many various methods can be used for the detection and determination of dopamine. All developed methods have some advantages and disadvantages, but SERS spectroscopy, due to its low limit of detection, was considered to be a very promising technique for carrying out such analyses. Moreover, in SERS measurements, it is possible to record a good quality spectrum even from a single molecule [21,22]; therefore, SERS spectroscopy is generally considered a very promising analytical tool. In our previous work, we showed that dopamine can also be very sensitively detected using SERS spectroscopy [18]. In these experiments, carried out using the film formed from plasmonic nanoparticles, we observed that the addition of NaCl significantly influences the measured SERS spectra of dopamine and significantly decreases the LOD of dopamine. In the case of experiments with sols of plasmonic nanoparticles, the addition of electrolytes may induce an aggregation of the nanoparticles and, hence, may induce a significant change in the intensity of the recorded SERS spectra [23,24,25]; the mechanism of the observed effect was unclear. Therefore, in this work, we decided to carry out SERS measurements using macroscopic nanostructured silver substrates, with all silver nanograins strongly connected to the substrate.

The determination of the orientation of the molecules of dopamine versus the silver surface was carried out using SERS surface selection rules and assignments of the Raman bands of dopamine, which were previously obtained in density-functional theory (DFT) simulations [26,27]. DFT is a quantum chemical theory in which the electronic charge density is the basic quantity used to determine the ground state properties of a many-body quantum system. First, the optimized geometrical structure corresponding to the minimum on the potential energy surface has to be obtained [27,28,29,30], and then harmonic vibrational frequencies are calculated using analytical second-order derivatives [27]. To obtain the assignments of the Raman bands, the polarizability derivatives, with respect to the normal coordinates, have to be extracted.

## 2. Materials and Methods

### 2.1. Reagents and Chemicals

Sodium fluoride, sodium chloride, sodium bromide, sodium iodide, sodium sulfate(VI), and dopamine hydrochloride were purchased from Sigma-Aldrich Poland (Poland, Poznań). Potassium chloride and ethanol were acquired from Chempur (Poland, Gliwice). All of the reagents mentioned above were used without further purification. The water was purified using a Millipore Milli-Q system (Merck Millipore, Burlington, MA, USA). The silver plates were acquired from Mennica Polska (Poland, Warsaw).

### 2.2. Preparation of Silver Substrates for SERS Measurements

The nanostructured silver substrates for the SERS experiments were prepared by electrochemical roughening of silver plates. The silver electrodes were obtained by cutting 10 × 5 mm rectangles from the silver sheet. Immediately before carrying out the electrochemical nanostructuring, the surface of the silver electrodes was sanded with sandpaper to remove the outer oxidized layer, and the electrodes were then thoroughly washed with ethanol and water. When placed in an electrochemical cell, only the fragment of the electrode with dimensions of 7 × 5 mm was immersed in the electrolyte. The electrochemical roughening was carried out in a three-electrode system (working electrode–silver plate; auxiliary electrode–platinum plate; reference electrode–Ag/AgCl/1.0 M KCl electrode). The primary electrolyte used was a 0.1 M KCl solution. First, three successful positive–negative–positive cycles were performed from –0.3 V to 0.3 V to –0.3 V. The sweep rate was 5 mV/s. Then, the silver electrode was kept at –0.4 V for 5 min to reduce all of the remaining AgCl.

### 2.3. Experimental Techniques

The Microlab 350 (Thermo VG Scientific, Waltham, MA, USA) microscope was used for the scanning electron microscopic (SEM) morphological characterization of the surface of the nanostructured silver.

The electrochemical roughening of the silver electrodes was performed in a conventional three-electrode cell using a PGSTAT204 potentiostat/galvanostat (Metrohm Autolab BV, NOVA 1.10 software).

All Raman measurements were performed using a Horiba Jobin-Yvon Labram HR80 (Palaiseau, France) spectrometer equipped with a Peltier cooled charge-coupled device detector (1024 × 256 pixels), a 600 groove/mm holographic grating, and an Olympus BX40 microscope (Shinjuku, Japan) with a long-distance 50× objective. A Nd:YAG laser provided the excitation radiation, with a wavelength of 532 nm; the power of the laser beam on the laser head was 70 mW.

The SERS measurements were carried out for the SERS silver substrates immersed in a small vessel into which 1 mL of an appropriate solution (pure water for the control experiments, dopamine solution, dopamine + salt solution, or only salt solution) was introduced. The dopamine solutions (for each measurement, 5 mL of the solution was prepared) were obtained by the appropriate diluting of a dopamine hydrochloride solution with a concentration of 0.1 M. In the case of the measurements in the electrolyte solution, an appropriate amount of salt was introduced into 5 mL of dopamine solution or into 5 mL of water.

## 3. Results

### 3.1. SEM Characterization of the SERS Substrates

**Figure 2:**
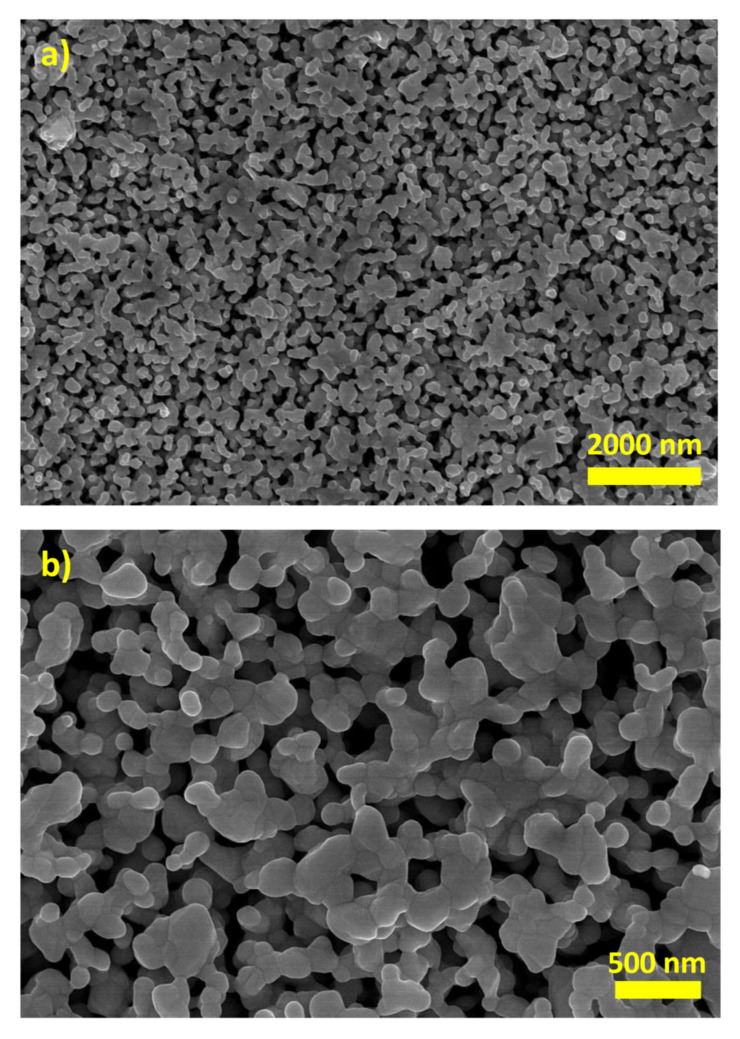
Scanning electron microscopy (SEM) images of the roughened silver electrode at two different magnifications (**a**,**b**).

The SERS measurements were made on the surface of a macroscopic nanostructured silver electrode (the nanostructuring was carried out by electrochemical oxidation–reduction cycling; for details see Section 2.2) instead of using a sol of silver nanoparticles (or films formed from silver nanoparticles, also typically used for SERS measurements) because the addition of salts might cause an aggregation of the colloidal plasmonic nanoparticles, which can significantly change the SERS enhancement factor [23,24,25]. The morphological characterization of the formed nanostructured silver was performed by a scanning electron microscope, and the obtained image is shown in Figure 2.

### 3.2. SERS Spectrum of Dopamine Recorded before the Addition of Electrolytes

**Figure 3:**
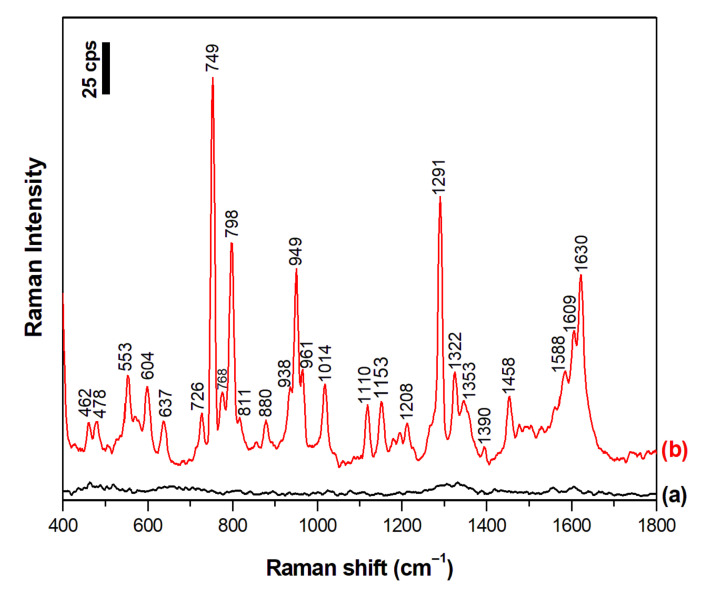
SERS spectrum of (a) the nanostructured silver electrode before the adsorption of dopamine and (b) the nanostructured silver electrode immersed in a 10^−5^ M aqueous solution of dopamine. Excitation wavelength: 532 nm. Accumulation time: 40 s. The spectra was averaged from 10 measurements.

The experiments were started by recording the SERS spectra of dopamine (the structural formula of dopamine is shown in Figure 1) adsorbed on the nanostructured silver surface from a 10^−5^ M dopamine aqueous solution to which no electrolytes had been added (the recorded SERS spectrum is presented in Figure 3). The SERS spectrum of dopamine recorded in such a condition is dominated by five strong Raman bands at 749 cm^−1^, 798 cm^−1^, 949 cm^−1^, 1291 cm^−1^, and 1630 cm^−1^ (see Figure 3). The first three bands (at 749 cm^−1^, 798 cm^−1^, and 949 cm^−1^) have been assigned to various C–H out-of-plane bending; whereas, the two other bands (at 1291 cm^−1^ and 1630 cm^−1^) are due to relatively complex ring vibrations [26,27]. The proposed assignments of the Raman bands of dopamine based on the DFT calculations carried out by Ciubuc et al. [26] and by Yadav and Mukherjee [27] are presented in Table 1.

**Figure 4:**
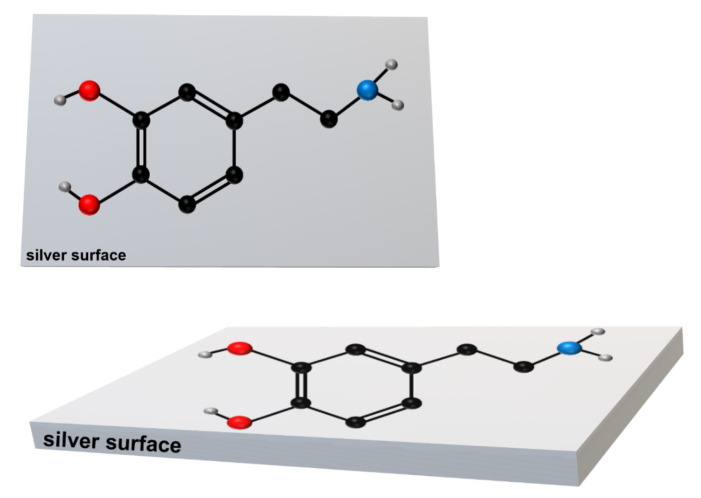
Schematic drawing of the dopamine molecule adsorbed on the silver surface from the aqueous solution to which no additional electrolytes have been added. The aromatic ring of dopamine adopts a flat orientation versus the silver surface. Upper drawing: view from above; bottom drawing: side view.

In SERS spectroscopy, the very large increase in the efficiency of the generation of the Raman signal is explained by the synergistic cooperation of the following two mechanisms: (i) a local increase in the intensity of the electric field in close proximity to the illuminated plasmonic nanostructures due to the excitation of the localized surface plasmons, where the increase in the efficiency of the generation of the Raman spectrum is roughly proportional to the fourth power of such field enhancement [32,33], and (ii) the resonance-like Raman effect, leading to an increased cross section for Raman scattering for chemisorbed molecules. Since the gradient of the electric field along the metal surface is suppressed (the efficiency of the damping of the gradient of the electric field along the metal surface increases with the decreasing frequency of the fluctuating electric field), the first abovementioned mechanism of the SERS enhancement leads to the SERS surface selection rule that states that Raman bands due to normal modes with polarizability derivative components perpendicular to the metal surface will be the most enhanced in SERS spectroscopy [34,35]. Therefore, the appearance of the strong SERS bands due to the out-of-plane vibrations suggests that, at this condition, the molecules of dopamine adopt a flat orientation versus the silver surface. A schematic drawing of the proposed orientation of the dopamine molecule (in two different views) versus the silver surface when dopamine is adsorbed from the aqueous solution to which no additional electrolytes have been added, is shown in Figure 4.

### 3.3. The Influence of Electrolytes on the SERS Spectra of Dopamine

**Figure 5:**
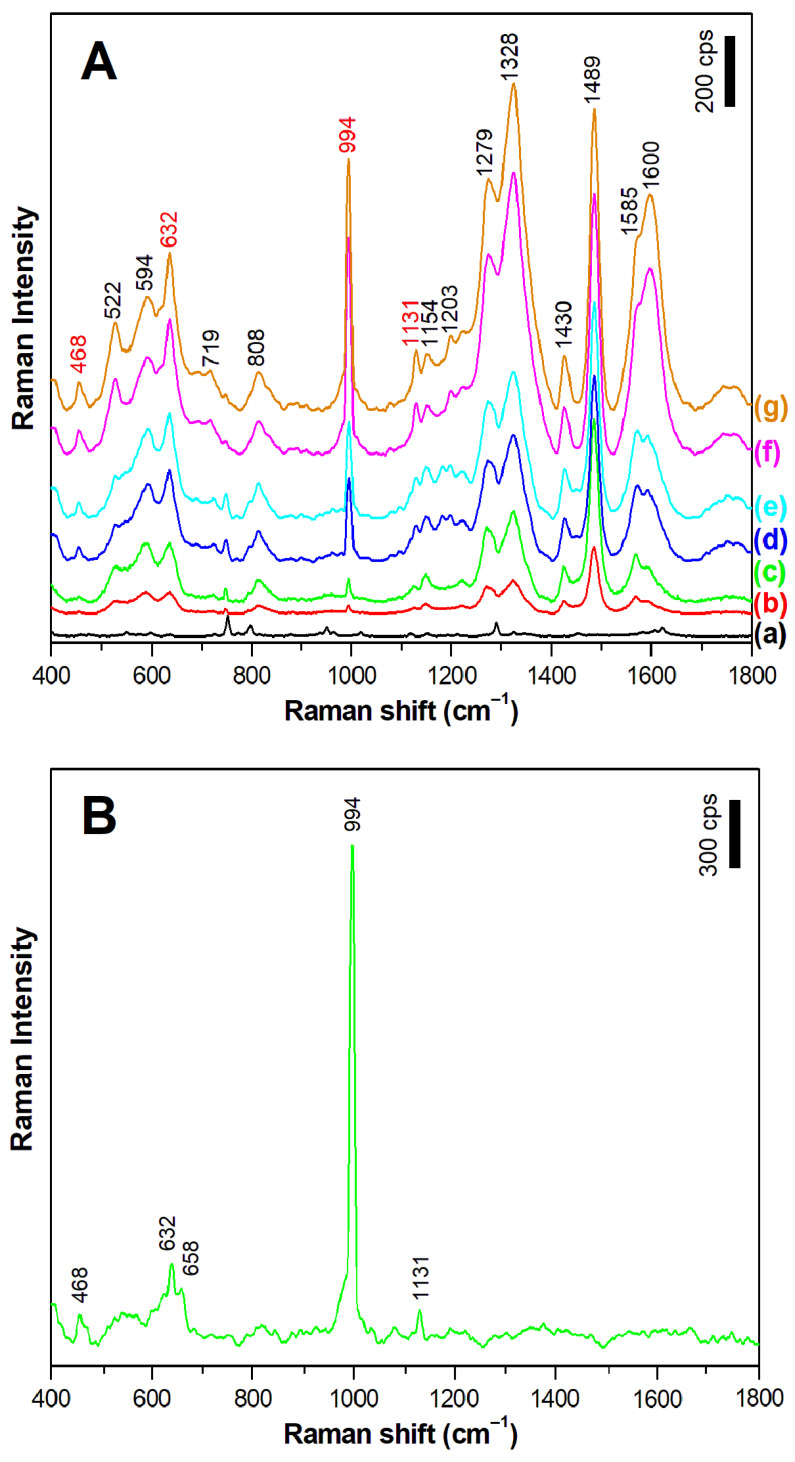
(**A**) SERS spectra of the dopamine adsorbed on the nanostructured silver electrode from a 10^−5^ M dopamine aqueous solution (a) before the addition of an electrolyte and (b–g) with the addition of Na_2_SO_4_. The final concentration of Na_2_SO_4_ is as follows: (b) 0.1 M, (c) 0.2 M, (d) 0.4 M, (e) 0.6 M, (f) 0.8 M, and (g) 1 M. The bands marked in red (at 468, 632, 994, and 1131 cm^−1^) are due to the vibrations of the SO_4_^2–^ anions. (**B**) SERS spectrum of SO_4_^2–^ anions adsorbed on the silver surface from a 1 M Na_2_SO_4_ aqueous solution. Excitation wavelength: 532 nm. Accumulation time: 40 s. The spectra was averaged from 10 measurements.

**Figure 6:**
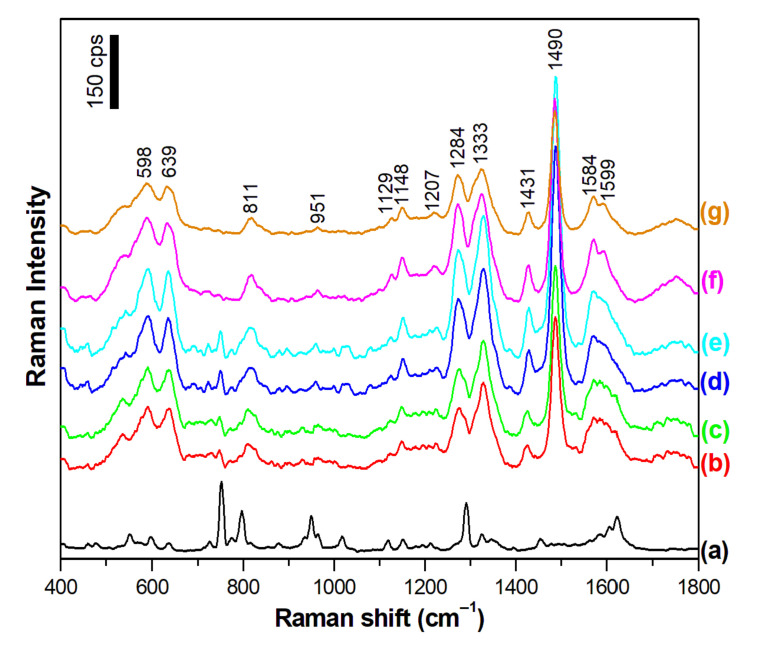
SERS spectra of the dopamine adsorbed on the nanostructured silver electrode from a 10^−5^ M dopamine aqueous solution (a) before the addition of an electrolyte and (b–g) with the addition of NaF. The final concentration of NaF is as follows: (b) 0.1 M, (c) 0.2 M, (d) 0.4 M, (e) 0.6 M, (f) 0.8 M, and (g) 1 M. Excitation wavelength: 532 nm. Accumulation time: 40 s. The spectra was averaged from 10 measurements.

Dopamine is usually administered as a drug in the form of a solution in saline (0.9% or 0.15 M aqueous solution of NaCl). Therefore, we decided to check how the presence of some electrolytes in the solution from which dopamine is adsorbed on the SERS substrates affects the intensity of the measured SERS spectrum. Figure 5 and Figure 6 show the SERS spectra of dopamine adsorbed on a silver surface from a 10^−5^ M dopamine aqueous solution to which various amounts of Na_2_SO_4_ and NaF have been added, respectively (similar experiments have also been carried out using the following electrolytes: NaCl, NaBr, and NaI). As it can be seen from the comparison of the spectra presented in Figure 4 and Figure 5, the addition of electrolytes induces a significant:

1. change in the spectral patters in the recorded SERS spectra;
2. change in the intensity of the recorded SERS spectra.

**Figure 7:**
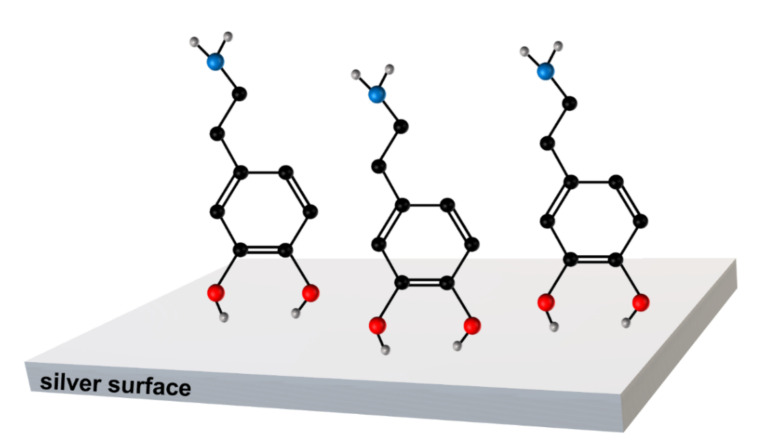
Schematic drawing showing the orientation of the dopamine molecules versus the silver surface when the dopamine is adsorbed from the solution containing an electrolyte.

A change in the spectral patterns of the recorded SERS spectra can be caused by a change in the chemical form of an absorbed molecule (in the case of dopamine, there is a possibility of a dissociation of the phenolic moieties or protonation of the amino group; see Figure 1) or it can be caused by a change in the orientation of the dopamine molecule versus the metal surface. Since the electrolytes used (e.g., NaCl) do not induce any significant changes in the pH of water, we suppose that the observed change in the spectral patterns is mainly due to the reorientation of the adsorbed molecules. The SERS spectra of dopamine, recorded in experiments when some electrolytes have been added, are dominated by bands mainly assigned to vibrations of a part of the dopamine molecule where two phenolic moieties are located, which are at 1489/1490 cm^−1^ due to a catechol ring breathing contributed mainly by the stretching of a carbon–carbon bond to which oxygens are attached (see Figure 4 and Figure 5) [31], and the bands at 1328/1333 cm^−1^ and 1279/1284 cm^−1^, which involve vibrations with a significant contribution of carbon–oxygen stretching vibrations [31]. These suggest that when adsorbed from a solution containing an electrolyte, dopamine interacts with the metal surface mainly via the phenolic moieties. Moreover, all of the strongest bands in the SERS spectra recorded at such conditions are due to in-plane vibrations. It is possible to state, according to the SERS surface selection rules [34,35], that at these conditions, the dopamine molecule adopts a perpendicular orientation versus the surface of the silver electrode. The possible orientation of the adsorbed dopamine molecules versus the silver surface is presented in Figure 7.

**Figure 8:**
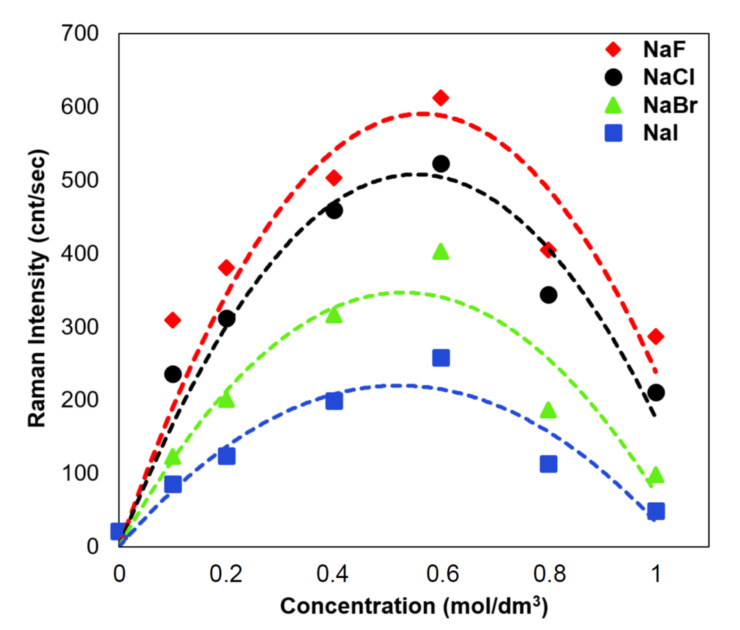
The dependence of the intensity of the dopamine band at 1490 cm^−1^ as a function of the concentration of introduced halides: (■) NaI, (▲) NaBr, (●) NaCl, and (♦) NaF.

As it can be seen from Figure 5 and Figure 6, the intensity of the measured SERS spectrum of dopamine strongly depends on the concentration of the added electrolytes. We carried out a deeper analysis of this effect using solutions of various halides (NaF, NaCl, NaBr, and NaI). Figure 8 shows the dependence of the intensity of the dopamine band at 1490 cm^−1^ (an example of one of the strongest bands in the recorded SERS spectra) as a function of the concentration of the introduced halides. As it can be seen from Figure 8, the most intense SERS spectra are recorded when the concentration of the introduced halides is equal to about 0.6 M. An increase in the concentration of the halides above this value leads to a decrease in the intensity of the measured SERS spectra, which is probably due to competition in the adsorption of the dopamine molecules and halides. When the concentration of the halides increases, their surface concentration also increases, and, hence, the surface concentration of dopamine decreases. For low concentrations of halides, an increase in their concentration leads to an increase in the intensity of the recorded SERS spectrum of dopamine. This effect is probably due to the anion-induced reorientation of the dopamine molecules and a larger value of the respective component of the polarization tensor for the dopamine molecule. It is also possible that for complexes formed from dopamine molecules and halide anions, a larger SERS enhancement due to the resonance-like effect (see Section 3.2) is generated compared to dopamine molecules that do not form such complexes. This would also explain the different halide-induced enhancements generated by various anions (the largest for fluorides and the smallest for iodides). However, other explanations of the observed phenomenon are also possible, for example, that iodides most effectively block the silver surface and, hence, the surface density of dopamine decreases in the experiments with NaF, NaCl, NaBr, and NaI, respectively. No matter what the mechanism of this phenomenon is, it can be stated that the addition of electrolytes has a very strong influence on the recorded SERS spectrum of dopamine, and this effect should be taken into account during interpretations of the results obtained with dopamine SERS sensors.

## 4. Conclusions

Dopamine is one of the most important neurotransmitters in the human body, and disturbed concentration levels of dopamine have been connected to various neurological disorders, such as Parkinson’s disease, Alzheimer’s disease, or schizophrenia. Therefore, the sensitive detection of this neurotransmitter is necessary, for instance, in the diagnostics of diseases or in the control of medical treatments. One of the most sensitive techniques for the detection and determination of dopamine is SERS spectroscopy [18]. In this contribution, we report a very strong influence of some salts on the spectral patterns and intensity of the recorded SERS spectra of dopamine adsorbed on a nanostructured macroscopic silver substrate. The analysis of the recorded SERS spectra is based on the SERS surface selection rules and on the assignments of Raman bands from previously reported DFT calculations. The carried out experiments revealed that if the molecules of dopamine are adsorbed from aqueous solutions into which no electrolytes have been introduced, they adopt a flat orientation versus the silver surface; whereas, if the molecules of dopamine are co-adsorbed with ions, they interact with the silver surface mainly via phenolic groups and adopt a perpendicular orientation versus the metal surface. The addition of electrolytes also significantly influences the intensity of the recorded SERS spectrum. For example, the addition of Na_2_SO_4_ to a final concentration of 1 M induces an increase in the intensity of the measured SERS spectrum by a factor of ca. 40. The abovementioned dependences should be taken into account during the interpretation of the results obtained with the dopamine SERS sensors (in many cases, the addition of electrolytes to the analyzed solution can reduce the limit of detection of dopamine by SERS spectroscopy).

## Figures and Tables

**Table 1:** Assignments of the SERS bands of dopamine based on the DFT calculations (according to refs. [26,27,31]).

Assignment	Raman Shift [cm^−1^]
C-O out of plane bending	462
C-C twisting	478
C-C twisting	553
C-H in-plane	604
C-C twisting	637
C-C twisting	726
C-H out-of -plane	749
CH_2_ twisting	768
C-H out-of -plane	798
NH_2_ wagging	811
C-C stretching	880
N-H twisting	938
C-H out-of-plane bending	949
C-C twisting	961
CH_2_ rocking	1014
NH_2_ rocking	1110
O-H rocking	1153
CH_2_ wagging/C-O stretching	1208
the catechol C-O stretching	1270
ring vibration involving C-H aromatic rocking and C-H twisting	1291
C-H in-plane bending	1322
C-O bond stretching	1335
C-C stretching/O-H scissoring	1353
C-H wagging/N-H twisting	1390
C-C stretching	1458
catechol ring breathing contributed mainly from the stretching of the C-C bond to which the oxygens are attached	1490
NH_2_ scissoring/CC stretching	1588
ring deformation	1609
ring deformation/NH_2_ scissoring	1630

## Data Availability

Not applicable.
